# Anxiety as a Common Biomarker for School Children With Additional Health and Developmental Needs Irrespective of Diagnosis

**DOI:** 10.3389/fpsyg.2019.01420

**Published:** 2019-06-21

**Authors:** Alana Jade Cross, Nahal Goharpey, Robin Laycock, Sheila Gillard Crewther

**Affiliations:** School of Psychology and Public Health, La Trobe University, Melbourne, VIC, Australia

**Keywords:** anxiety, autism spectrum disorder, autism traits, sleep, language impairment, learning difficulties, additional needs children

## Abstract

Currently very little evidence is available regarding the biological characteristics and common comorbid behaviors that are associated with children characterized by learning difficulties who require additional support at school. These children are usually referred to as having Additional Health and Developmental Needs by the Australian Government and the associated public education system more broadly though the problems may arise from academic, social and/or emotional stressors and may or may not include children with clinically diagnosed Neurodevelopmental Disorders. Thus, the aim of this study was to investigate the relationship between anxiety levels (Spence Children’s Anxiety Scale- Parent Report), autism traits (Autism Spectrum Quotient – Child Version) and sleep quality (Sleep Disturbance Scale for Children) in children with Additional Health and Developmental Needs without an intellectual disability, but with either a diagnosis of Autism Spectrum Disorder (ASD) (*N* = 25), Speech and Language Impairment (*N* = 37) or Other Diagnosis (*N* = 22). Our results demonstrated that these children with Additional Health and Developmental Needs showed atypically high levels of anxiety and impaired sleep quality, with the ASD group reporting more impairments associated with comorbid anxiety and sleep quality than either of the other clinically diagnosed groups. In fact, greater anxiety level was associated with a greater number of autism traits and poorer sleep quality regardless of diagnostic group suggesting that anxiety is a common experience for children with Additional Health and Developmental Needs. It is suggested that assessment of anxiety, sleep behaviors and autism traits may be useful markers for early identification of children within this population, thus providing scope for early and targeted intervention.

## Introduction

Childhood is the most critical developmental period (Irwin et al., 2007), making the identification of educational and mental health difficulties and the need for developmentally appropriate early intervention fundamental (Noam and Hermann, 2002; Atkins et al., 2017). It is estimated that at least 1 in 5 children in Australia require extra support at school due to a wide range of diagnosed and undiagnosed developmental disorders, learning, behavioral and/or emotional difficulties (Goldfeld et al., 2012). These children are classified by the Australian Government and public education system as having Additional Health and Developmental Needs (AHDN) (Howell-Meurs et al., 2014; O’Connor et al., 2015), sometimes referred to as Special Health Care Needs (Bethell et al., 2002; O’Connor et al., 2019). Thus AHDN children have come to be categorized as “*those who have, or are at increased risk for a chronic physical, developmental, behavioral, or emotional condition and who also require health and related services of a type or amount beyond that required by children generally”* (McPherson et al., 1998). Such needs may be established (diagnosed at school entry) or emerging (identified by teachers) (Goldfeld et al., 2015), suggesting that difficulties noted at school may be indicative of an underlying biological problem. The developmental characteristics of children with AHDN are largely unknown, given that they are often excluded from research, government policies and access to funding for interventions (McDowell and O’Keeffe, 2012; Dempsey and Davies, 2013).

We set out to investigate whether such a group of children with AHDN share common biological characteristics and behavioral comorbidities irrespective of the etiology or environmental source of their primary difficulty. Anecdotally it is often assumed that learning difficulties are a source of anxiety, a claim that is supported by evidence that lower intellectual ability is related to increased anxiety, sleep difficulties and unsuccessful social interactions (Rzepecka et al., 2011). Lower childhood IQ is also known to be associated with an increased risk of developing Generalized Anxiety Disorder (GAD), obsessive–compulsive symptoms, depression and schizophrenia in adulthood (Martin et al., 2007; Koenen et al., 2009; Grisham et al., 2011). Conversely, higher childhood IQ can be a protective factor for anxiety, with performance one standard deviation above the mean on a Full Scale IQ assessment at the age of seven thought to reduce the lifetime risk of a GAD diagnosis by 50% (Martin et al., 2007). Thus, this study focused on investigating anxiety in children with a range of diagnoses who were identified as struggling at school.

Anxiety is a universal human experience, underpinning basic adaptive behaviors such as the ‘fight, flight or freeze’ response (Bracha, 2004). By comparison, diagnosed or clinical childhood anxiety is a leading burden of disease (World Health Organisation [WHO], 2013b; Baranne and Falissard, 2018) and is associated with poor self-esteem and decreased quality of life, with even subthreshold symptoms being predictive of clinical anxiety, depression and mood disorders in both adolescence and adulthood (Kovacs and Lopez-Duran, 2010; Rapee, 2012; van Os, 2013; Martinsen et al., 2016).

Given that anxiety is known to affect an individual’s entire biological system, we further propose that certain behaviors may be symptomatic of anxiety (core biomarkers) (Shadli et al., 2015; Maron and Nutt, 2017; Meyer, 2017), such as insomnia and worry (prolonged rumination about future events) (McGorry, 2013; van Os, 2013). These behaviors may predict behavioral expressions of anxiety (such as sleep and autism traits) in young children who do not necessarily meet full diagnostic classification of an Anxiety Disorder but still have deficits in everyday functioning. Identification of core biomarkers is especially important for children who may not be able to verbally express their anxiety or identify emotions due to difficulties with speech and language such as those with autism spectrum disorder (ASD) (Green and Ben-Sasson, 2010). Early intervention has been reported to reduce anxiety in children, however, longer term outcomes remain largely unknown in adolescence and adulthood (Barrett et al., 2006; Neil and Christensen, 2009; Werner-Seidler et al., 2017), highlighting the need for better detection earlier in childhood (Neil and Christensen, 2009; Bayer et al., 2011; Fisak et al., 2011).

Anxiety shares many core features and is commonly comorbid with ASD (Grondhuis and Aman, 2012; Kerns and Kendall, 2014), where anxiety has been suggested as an underlying cause of ASD symptoms (Green and Ben-Sasson, 2010; Gotham et al., 2013). An otherwise neurotypical child who presents with anxiety often exhibits a spectrum of behaviors consistent with ASD traits such as rigidity of thought, inflexible behavior, sleep difficulties and emotional dysregulation (van Steensel et al., 2011). Such observations have led to the conceptualization of the spectrum of autism as including non-clinical individuals in the wider population who possess related traits to varying degrees (Auyeung et al., 2008), and whose behavior is recognized as associated with anxiety, depression, worse educational performance (Rosbrook and Whittingham, 2010; Freeth et al., 2013) and sleep disturbances (Limoges et al., 2005).

Anxiety and sleep have a bidirectional relationship (Alvaro et al., 2013; Kahn et al., 2013). Sleep problems in childhood are associated with increased symptoms of Generalized Anxiety Disorder, somatic complaints, aggression, poorer empathy and decreased attention (Fischer et al., 2014; Simola et al., 2014; Brand et al., 2016; Fletcher et al., 2016; James and Hale, 2017) and are recognized as having negative consequences for learning and attention, reduced concentration and immune system functioning (Segerstrom and Miller, 2004; Curcio et al., 2006). Difficulty with sleep can be a behavioral consequence of anxiety that is also associated with ASD symptoms (Schreck et al., 2004; Alfano et al., 2007; Veatch et al., 2017). Poor sleep is known to contribute to challenging behaviors across the autism spectrum (Richdale and Schreck, 2009; Rzepecka et al., 2011; Cohen et al., 2014, 2018; May et al., 2015) leaving us wondering if sleep disturbances might also be an important characteristic of children with AHDN. Thus as previous literature has documented a relationship between anxiety and sleep quality (Alfano et al., 2007), and anxiety and ASD (Vasa et al., 2014), we set out to investigate the relationships between all three variables of anxiety, autism behaviors and sleep quality in children with AHDN.

We have chosen to use parent answered questionnaires as an accepted way (Eisert et al., 1991) to identify common experiences of anxiety and associated autism traits and sleep behaviors in AHDN children. We also acknowledge that parents of children with disorders such as ASD are likely to be better at identifying overtly observable, rather than internally experienced, symptoms of anxiety due to the child’s difficulty expressing and accurately communicating emotions (Magiati et al., 2014). Thus, the aims of this study were to investigate the relationship between parent perceived childhood anxiety, sleep problems and autism traits across clinical diagnostic categories in AHDN children, and to identify which aspect of anxiety is most associated with sleep and autism-like behaviors. An associated aim of this study was to determine whether increased anxiety is associated with a Neurodevelopmental Disorder diagnosis.

We hypothesized that children who have high levels of parent rated anxiety will have greater parent reported severity of autism traits and sleep disturbance than children with lower levels of anxiety regardless of diagnosis. Given that we propose anxiety as a common biomarker in AHDN children, it is anticipated that our overall sample will have higher anxiety scores than normal controls found in previous research despite their differing diagnoses (Nauta et al., 2004). Furthermore, we anticipated that children with a diagnosis of ASD would have even greater levels of parent reported anxiety compared to other groups, consistent with past research reporting high comorbidity (van Steensel et al., 2011).

## Materials and Methods

### Participants

A total of 90 children aged between 5 and 13 years participated in this study (see Table 1 for demographics). Participants were recruited from a school holiday therapy program for children experiencing social, emotional and academic difficulties in Melbourne, VIC, Australia and were identified as having AHDN due to requiring more support than their peers (McPherson et al., 1998). Inclusion criteria for the study aligned with children attending the therapy program and included clinical diagnoses of Attention Deficit Hyperactive Disorder (ADHD); Auditory Processing Impairments and Disorders; Communicative Social Deficits; Confidence and Self- Esteem Difficulties; ASD; Language Delay; Language Impairment; Language Disorders; Specific Language Impairment; Anxiety; Reading Delay, Impairment or Disorder; Dyslexia, Semantic and Pragmatic Disorders; Sensory Processing Disorders; Speech Sound Disorders and Dyspraxia; Spelling Delay or Impairment; Writing Delay, Impairment or Disorder; and Dysgraphia. Children with physical disability, intellectual disability, medical issues, or major mental health concerns related to trauma were excluded.

**TABLE 1:** Demographic variables of current sample (*N* = 84).

			***N***	**%**
Age	*M* (SD)	9.22 (1.85)	–	–
	Range	5 – 13	–	–
Gender	Female	–	26	31.00
	Male	–	58	69.00
Primary diagnosis	ASD	–	25	29.80
	Speech and language	–	37	44.00
	Other diagnosis	–	22	26.20
Co-morbid diagnoses	*M* (SD)	1.48 (1.16)	–	–
	Range	0 – 5	–	–

As the majority of children had been previously diagnosed with ASD or a Speech and Language disorder, the remaining children were categorized as a miscellaneous ‘Other’ diagnostic group for the purpose of statistical analyses. The ‘Other’ diagnostic group was created to include participants in the analysis who had a minority diagnosis and consisted of children with a reported primary diagnosis of ADHD (7 participants), a chromosomal condition (2 participants) or who were undiagnosed, however, experienced difficulties with social skills (13 participants). Children had up to 5 comorbid diagnoses from various health professionals, with an average of 1.48 comorbidities. Seven children were diagnosed with a comorbid anxiety disorder. Six participants were excluded as they were deemed to have an intellectual disability on the Ravens Colored Progressive Matrices screening measure of non-verbal intelligence, leaving a total sample 84. Parents were made aware of the aims of the study prior to providing written consent for participation. Written informed parental consent was obtained for all participants. This study was conducted with approval from the La Trobe University Human Ethics Committee and the Victorian Department of Education Human Ethics Committee.

Our sample was compared to data previously published by Nauta et al. (2004). Their Anxiety Group (*N* = 484) consisted of 220 females (45%) and 264 males (55%) aged 6–17 years (*M* = 10.4, *SD* = 2.5) who had between 0 and 5 co-morbid diagnoses (*M* = 1.6, *SD* = 1.3). By comparison, their control group (*N* = 261) had 136 females (52%) and 125 males (48%) aged between 6 and 18 years (*M* = 11.5, *SD* = 2.0).

### Materials

#### Screening Measures

Children were screened for normal or corrected to normal vision and hearing and assessed to exclude intellectual disability on the Raven’s Colored Progressive Matrices (RCPM). The RCPM is a widely used measure of fluid intelligence in children (Raven et al., 1990), and consists of 36 incomplete matrix puzzles (three sets of twelve) of increasing difficulty. Children were asked to identify the missing piece of the puzzle from six possible segments in order to best complete the matrix design. The task was not time limited.

#### Anxiety

Anxiety was assessed using the Spence Children’s Anxiety Scale- Parent Version (SCAS-P) (Nauta et al., 2004). The SCAS-P is a 38-item questionnaire consisting of six subscales: ‘Panic Attack and Agoraphobia,’ ‘Separation Anxiety,’ ‘Physical Injury Fears,’ ‘Social Phobia,’ ‘Obsessive Compulsive,’ and ‘Generalized Anxiety Disorder.’ Parents were asked to rate each statement in terms of the frequency of an anxiety-related behavior observed in their child from ‘Never,’ ‘Sometimes,’ ‘Often’ or ‘Always’ with no specified time period. These responses were scored on a Likert scale from 0 (‘Never’) through to 3 (‘Always’) with a maximum possible score of 144. Higher scores indicated greater parent perceived childhood anxiety.

#### Autism Traits

Autism traits were measured using the Autism Spectrum Quotient- Child Version (AQ-Child) (Auyeung et al., 2008). The AQ-Child is a 50-item parent report questionnaire examining autism-like behaviors in five subscales: ‘Social Skills,’ ‘Attention Switching,’ ‘Attention to Detail,’ ‘Communication,’ and ‘Imagination.’ Each question asked parents to rate the degree their child exhibited autism-like behaviors by responding ‘Definitely Agree,’ ‘Slightly Agree,’ ‘Slightly Disagree,’ or ‘Definitely Disagree.’ Responses were scored from 0 (‘Definitely Agree’) to 3 (‘Definitely Disagree’) to a maximum score of 150, with higher scores indicating higher level of autism-like traits.

#### Sleep

Sleep behaviors were assessed using the Sleep Disturbance Scale for Children (SDSC) (Bruni et al., 1996). The SDSC is a 27-item questionnaire for parents to rate their child’s sleep pattern over the past 6 months across six subscale domains: ‘Disorders of Initiating and Maintaining Sleep,’ ‘Sleep Breathing Disorders,’ ‘Disorders of Arousal,’ ‘Sleep Wake Transition Disorders,’ ‘Disorders of Excessive Somnolence,’ and ‘Sleep Hyperhydrosis.’ Questions were rated from ‘Never,’ ‘Occasionally’ (up to once or twice per month), ‘Sometimes’ (once or twice per week), ‘Often’ (three to five times per week) to ‘Always’ (daily). These responses were scored on a Likert scale from 0 (‘Never’) to 5 (‘Always’) with a maximum total score of 130. Higher scores were associated with increased sleep disturbance.

### Procedure

At the commencement of the 3-week school holiday program, parents were greeted by the researchers and provided with a verbal and written explanation of the research. Informed written consent was obtained prior to the commencement of data collection. Parents were invited to complete pen and paper versions of the AQ-Child, SCAS-P and SDSC about their child as well as a demographics questionnaire asking for the child’s medical history and any clinical diagnosis, school grade and age. Parents were asked to complete and return all questionnaires within a 1-week timeframe. All participants were screened for visual and hearing impairments. A subsample of participants completed the RCPM where time was permitted amongst other activities at the holiday program.

### Data Analysis

All data was entered into the computer program SPSS (Version 23) and descriptive statistics were performed on all variables. Data screening was conducted for all measures to inspect for missing data, outliers and potential violations to assumptions of normality and homogeneity. Missing data was excluded from the analyses. The data was assessed using standard skewness and kurtosis measures, the Kolmogorov–Smirnov, and the Shapiro–Wilko test. These results indicated that the assumption of normality was violated, as expected given the atypical sample used in this study. Therefore, a non-parametric approach to data analysis was used. A Levene’s test revealed assumptions of homogeneity were also violated.

Participants were split into High and Low Anxiety groups associated with the top and bottom third of SCAS-P total score, and their means and standard deviations compared to previously published scores for Anxiety Disordered and Normal Control children. A Kruskall’s Wallis analysis was performed in order to examine any difference between diagnostic groups. The relationship between all variables was assessed using Spearman’s Rho.

### Relationship With Non-verbal Intelligence

A subsample of participants *(N* = 48) completed the Raven’s Standard Progressive Matrices. A Spearman’s rank order correlation analysis found no significant relationship between Raven’s performance and anxiety (SCAS-P) total score, *r_s_*(46) = 0.08, *p* = 0.58. The relationship between Raven’s performance and total sleep (SDSC) score approached significance, *r_s_*(46) = 0.28, *p* = 0.06. A weak to moderate significant relationship was found between Raven’s Standard Score and autism traits (AQ-Child) total score *r_s_*(46) = 0.35, *p* = 0.01.

## Results

### Descriptive Statistics on Anxiety

Descriptive statistics associated with the sample and their scores on the SCAS-P total and subscales, as a measure of overall anxiety in the current sample are recorded and compared to previously published data (Nauta et al., 2004) in Table 2. Comparison of groups demonstrates that the participants in our study had higher levels of anxiety compared to normal controls from previous research (Cohen’s *d* = 0.86). The High Anxiety group demonstrated moderately higher anxiety than the Nauta et al. (2004) anxiety disordered group (Cohen’s *d* = 0.44), whilst the Low Anxiety group demonstrated a smaller effect with lower anxiety compared with the normal controls from Nauta et al. (2004) (Cohen’s *d* = 0.34).

**TABLE 2:** Means and standard deviations for anxiety (Spence, SCAS-P) for current sample and published data.

	**The current sample**			**Published data (Nauta et al., 2004)**	
**SCAS-P Subscale**	**Total sample**	**High anxiety**	**Low anxiety**	**Anxiety disordered**	**Normal controls**
	**(*N* = 84)**	**(*N* = 28)**	**(*N* = 28)**	**(*N* = 484)**	**(*N* = 261)**
Age (*M*, *SD*)	9.22 (1.85)	9.41 (1.59)	9.29 (2.34)	10.4 (2.5)	11.5 (2.0)
Ravens	*N* = 48	*N* = 14	*N* = 17	–	–
	107.35 (10.02)	105.71 (12.75)	107.47 (8.60)		
Separation anxiety	4.86 (3.39)	8.29 (2.90)	1.82 (1.34)	6.9 (4.1)	2.6 (2.8)
Generalized anxiety	4.51 (2.91)	7.79 (2.29)	1.89 (1.25)	6.6 (3.1)	2.7 (2.0)
Social phobia	6.14 (3.66)	8.71 (3.45)	3.71 (2.89)	7.7 (3.8)	4.2 (2.8)
Panic/Agoraphobia	1.80 (2.10)	3.64 (2.56)	0.50 (1.04)	3.6 (3.9)	1.0 (1.6)
Physical injury fears	3.85 (2.65)	5.82 (2.87)	2.11 (1.45)	4.1 (2.8)	2.6 (2.3)
Obsessive compulsive	2.12 (2.13)	3.71 (2.56)	1.00 (1.22)	3.0 (3.1)	1.1 (1.7)
Total	23.27 (12.78)	37.96 (9.80)	11.04 (4.05)	31.8 (14.1)	14.2 (9.7)

*Published data reported only to one decimal place. Ravens scores indicated are standardized (Cotton et al., 2005).*

### Total Scores by Diagnostic Category

Descriptive statistics for total SCAS-P, AQ-Child and SDSC and scores for the ASD (*N* = 25), Speech and Language (*N* = 37), and Mixed Diagnosis (*N* = 22), groups can be found in Table 3.

**TABLE 3:** Total scores of anxiety, autism traits, sleep and Ravens by sample diagnostic categories.

	**ASD (*N* = 25)**		**Speech and language (*N* = 37)**		**Mixed diagnosis (*N* = 22)**		**Total sample (*N* = 84)**	
	***M* (*SD*)**	**Range**	***M* (*SD*)**	**Range**	***M* (*SD*)**	**Range**	***M* (*SD*)**	**Range**
SCAS-P	29.76 (12.17)	9 – 53	19.35 (11.65)	3 – 59	22.50 (12.89)	7 – 52	23.27 (12.78)	3 – 59
AQ-Child	91.28 (13.73)	62 – 115	55.35 (16.08)	22 – 99	60.95 (24.36)	27 – 122	67.51 (23.75)	22 – 122
SDSC	48.72 (10.07)	33 – 71	39.92 (7.64)	26 – 58	44.00 (10.38)	32 – 72	43.61 (9.79)	26 – 72
Ravens	*N* = 14	94 – 123	*N* = 21	89 – 120	*N* = 13	94 – 125	*N* = 48	89 – 125
	112.36		102.14		110.38		107.35	
	(8.62)		(8.78)		(9.87)		(10.02)	

*Ravens scores indicated are standardized (Cotton et al., 2005).*

### Diagnostic Group Comparison on Anxiety, Autism Traits, Sleep

A Kruskall–Wallis *H* Test was run to determine if there were differences in anxiety (SCAS-P), autism traits (AQ-Child) and sleep behaviors (SDSC) between the three diagnostic groups. Where significant group differences were established, *post hoc* pairwise comparisons were performed using Dunn’s (1964) procedure with a Bonferroni correction for multiple comparisons. The distribution of questionnaire scores were similar for all diagnostic groups, as assessed by visual inspection.

#### Anxiety

Median Anxiety (SCAS-P) total scores were statistically significant between groups, χ^2^(2) = 11.76, *p* = 0.003. *Post hoc* analysis revealed statistically significant differences in SCAS-P total score between the ASD (*Mdn* = 28.00) and the Speech and Language (*Mdn* = 17.00) diagnostic groups (*p* = 0.002). There was no statistically significant difference in SCAS-P total score between the ASD and Mixed Diagnosis (*Mdn* = 20.00) groups, or Speech and Language and Mixed Diagnosis groups combinations.

#### Autism Traits

Median Autism Traits (AQ – Child) total scores were statistically significant between groups, χ^2^(2) = 37.85 *p* < 0.001. *Post hoc* analysis revealed statistically significant differences in AQ – Child total score between the ASD (*Mdn* = 92.00) and Speech and Language (*Mdn* = 56.00) diagnostic groups (*p* < 0.001), and ASD and the Mixed Diagnosis (*Mdn* = 59.50) groups (*p* < 0.001). There was no statistically significant difference in AQ – Child total score between the Speech and Language and Mixed Diagnosis groups.

#### Sleep Behaviors

Median Sleep Behavior (SDSC) total scores were statistically significant between groups, χ^2^ (2) = 11.04, *p* = 0.004. *Post hoc* analysis revealed statistically significant differences in SDSC total score between ASD (*Mdn* = 49.00) and Speech and Language (*Mdn* = 40.00) diagnostic groups (*p* = 0.003). There was no statistically significant difference in SDSC total score between the ASD and Mixed Diagnosis (*Mdn* = 42.50), or the Speech and Language, or Mixed Diagnosis groups combinations.

### Relationship Between Anxiety, Autism Traits and Sleep Across Diagnoses

A series of Spearman’s Rho rank correlations were conducted in order to examine the relationship between anxiety (SCAS-P), autism traits (AQ-Child) and sleep (SDSC) questionnaires for all participants. Preliminary analyses showed all relationships to be monotonic, as assessed by visual inspection of scatterplots. Table 4 demonstrates that there was a moderate positive relationship between the SCAS-P total score and the AQ-Child total score, as well as SDSC total score. There was a weak positive relationship between SCAS-P total score and SDSC total score.

**TABLE 4:** | Correlation between total scores for anxiety (SCAS-P), autism traits (AQ – Child) and sleep (SDSC) for total sample (*N* = 84).

	**SCAS-P**	**AQ-Child**	**SDSC**
SCAS-P	–	0.37^∗∗^	0.44^∗∗^
AQ-Child		–	0.28^∗∗^
SDSC			–

*^∗∗^*p* < 0.01.*

#### Anxiety Scale (SCAS-P)

A Spearman’s rank order correlation was run to assess the relationship between SCAS-P subscales (Table 5). There was a strong positive correlation between Total SCAS-P score and Separation Anxiety, Generalized Anxiety, Social Phobia and Panic/Agoraphobia subscales. There was a moderate positive correlation between Total SCAS-P score and Physical Injury Fear and Obsessive Compulsive subscales.

**TABLE 5:** Intercorrelations of the Spence Children’s Anxiety Scale subscales for total sample (*N* = 84).

	**Separation**	**Generalized**	**Social**	**Panic/**	**Physical injury**	**Obsessive**	
**SCAS-P subscale**	**anxiety**	**anxiety**	**phobia**	**Agoraphobia**	**fears**	**compulsive**	**Total**
Separation anxiety	–	0.78^∗∗^	0.44^∗∗^	0.42^∗∗^	0.54^∗∗^	0.43^∗∗^	0.82^∗∗^
Generalized anxiety		–	0.50^∗∗^	0.60^∗∗^	0.55^∗∗^	0.48^∗∗^	0.88^∗∗^
Social phobia			–	0.42^∗∗^	0.25^∗∗^	0.30^∗∗^	0.68^∗∗^
Panic/Agoraphobia				–	0.42^∗∗^	0.48^∗∗^	0.73^∗∗^
Physical injury fears					–	0.20	0.63^∗∗^
Obsessive compulsive						–	0.59^∗∗^

*^∗∗^*p* < 0.01.*

#### Anxiety (SCAS-P) and Autism Traits (AQ-Child)

A Spearman’s rank order correlation was run to assess the relationship SCAS-P and AQ-Child Subscales (Table 6). There was a moderate positive correlation between the Obsessive Compulsive subscale and AQ-Child total score. The Obsessive Compulsive subscale had weak to moderate positive correlations with Social Skills, Attention Switching, Attention to Detail, Communication and Imagination subscales of the AQ-Child, as well as total score. There were weak positive correlations between Separation Anxiety, Generalized Anxiety, Social Phobia, Panic/Agoraphobia, and SCAS-P total score to AQ-Child total score.

**TABLE 6:** Correlation between Spence Anxiety Scale and Autism Spectrum Quotient for total sample (*N* = 84).

	**Social**	**Attention**	**Attention**			
**AQ-Child**	**skills**	**switching**	**to detail**	**Communication**	**Imagination**	**AQ-C total**
SCAS-P						
Separation anxiety	0.30^*^	0.30^*^	0.13	0.28^*^	0.17	0.30^∗∗^
Generalized anxiety	0.31^*^	0.37^∗∗^	0.07	0.27^*^	0.15	0.30^∗∗^
Social phobia	0.25^*^	0.27^*^	0.01	0.22	0.14	0.20
Panic/Agoraphobia	0.26^*^	0.28^*^	0.12	0.24^*^	0.05	0.26^*^
Physical injury fears	0.14	0.16	–0.05	0.09	0.08	0.12
Obsessive compulsive	0.51^∗∗^	0.52^∗∗^	0.29^∗∗^	0.53^∗∗^	0.25^*^	0.51^∗∗^
SCAS-P total	0.38^∗∗^	0.40^∗∗^	0.10	0.35^∗∗^	0.20	0.37^∗∗^

^*^p < 0.05, ^∗∗^*p* < 0.01.

#### Anxiety (SCAS-P) and Sleep (SDSC)

A Spearman’s rank order correlation was run to assess the relationship between subscales of the SCAS-P and SDSC (Table 7). The SDSC total score demonstrated weak to moderate positive correlations with Separation Anxiety, Generalized Anxiety, and Social, Panic / Agoraphobia, Physical Injury Fears, and Obsessive Compulsive subscales from the SCAS-P. There was a moderate positive correlation between SCAS-P total score and SDSC Total score.

**TABLE 7:** Correlation between Spence Anxiety Scale and Sleep Disturbance Scale for Children for total sample (*N* = 84).

							**SDSC**
**SDSC**	**DIMS**	**SBD**	**DA**	**SWTD**	**DOES**	**SHY**	**Total**
SCAS-P							
Separation anxiety	0.33^∗∗^	–0.01	0.21	0.26^*^	0.21	0.16	0.38^∗∗^
Generalized anxiety	0.29^∗∗^	0.05	0.22^*^	0.21	0.16	0.28^∗∗^	0.39^∗∗^
Social phobia	0.11	0.16	0.09	0.12	0.15	0.29^∗∗^	0.26^*^
Panic/Agoraphobia	0.20^*^	0.02	0.14	0.19	0.10	0.18	0.25^∗∗^
Physical injury fears	0.24^*^	0.11	0.05	0.12	0.13	–0.03	0.24^*^
Obsessive compulsive	0.43^∗∗^	0.09	0.20	0.20	0.34^∗∗^	0.14	0.42^∗∗^
SCAS-P total	0.35^∗∗^	0.06	0.19	0.25^*^	0.25^*^	0.21	0.44^∗∗^

^*^p < 0.05, ^∗∗^*p* < 0.01; DIMS, Disorders of Initiating and Maintaining Sleep; SBD, Sleep Breathing Disorder; DA, Disorders of Arousal; SWTD, Sleep Wake Transition Disorder; DOES, Disorders of Excessive Somnolence; SHY, Sleep Hyperhydrosis.

## Discussion

This study aimed to identify the relationship between anxiety, sleep problems and autism traits as a means of characterizing the commonality of anxiety in children with additional social, emotional and academic needs across varying clinically diagnosed disorders. A secondary aim was to identify if there was an aspect of anxiety that would be most frequently associated with sleep and autism-like behaviors in our population sample. This was achieved by comparing parent-rated responses on measures of autism traits (AQ-Child), anxiety (SCAS-P), and sleep behaviors (SDSC) given that sleep and autism traits may represent observable behaviors driven by anxiety. It was hypothesized that higher parent perceived anxiety levels would be associated with more severe autism traits and poorer sleep quality in all children with identified AHDN, regardless of individual diagnoses. We anticipated that our overall sample would be more anxious compared to typically developing children regardless of diagnosis. We also hypothesized that children with ASD would have even greater levels of parent perceived anxiety than other diagnostic groups.

Overall, we found that our sample demonstrated higher levels of anxiety compared to a normal control population (Nauta et al., 2004), supporting the hypothesis that anxiety would be a common feature in children with AHDN and in particular when greater levels of autism traits were evident. Conceptualizing anxiety as a continuum suggests that, despite this commonality, the experience of anxiety differs in individuals to a greater or lesser extent. Total scores on all measures were higher in our High Anxiety group compared to both Anxiety Disordered and Normal Control group overall (Nauta et al., 2004). However only scores on the Physical Injury Fears and Obsessive–Compulsive subscales were higher than an Anxiety Disordered population, highlighting these as particularly prominent areas of worry in the current sample. Comparatively, our Low Anxiety group scores were lower than Normal Controls across all SCAS-P subscales which may be attributed to individual differences in the sample, varied parent’s perception, or measurement of anxiety at a trait rather than state level. These findings suggest that children with AHDN are a population at an increased risk of experiencing generally high levels of anxiety, and thus anxiety screening should form an integral part of assessment for teachers and mental health professionals.

Comparison between the ASD, Speech and Language, and Other Diagnosis groups on anxiety, autism and sleep measures revealed some differences between groups. The ASD group had significantly higher anxiety, autism traits and sleep difficulty when compared to the Speech and Language group, indicating that anxiety and sleep problems may be more pronounced in this group. As expected, the AQ –Child questionnaire also differentiated the ASD and Other Diagnosis groups. Despite differences noted in the ASD group, a key finding was that there was no significant difference in anxiety and sleep behaviors between the ASD and Other Diagnosis groups, or the Speech and Language and Mixed Diagnosis groups. It is important to note that high levels of anxiety were reported across groups, and that autism traits predicted anxiety and sleep difficulties regardless of diagnosis. While diagnostic classification may be useful in differentiating some aspects of behavior, the relationship between anxiety, sleep and autism traits may ultimately contribute to a broader neurodevelopmental phenotype in children with AHDN.

A number of other factors challenged the usefulness of diagnoses in children in the current study. Questionnaire scores for each diagnostic category showed very large ranges, highlighting the potential of previous professional misdiagnosis due to either inaccurate parent reporting or variation in standardized testing and diagnosis across different medical and allied health professionals. One example is that the highest recorded score on the AQ-Child was in the Mixed Diagnosis group rather than the ASD group. Given that all children experienced social, emotional and academic difficulties, such variation highlights the importance of recognizing individual differences and functional impairment for the child and their family beyond a diagnostic label.

Correlational analyses allowed a further examination of the relationship between behavioral traits. Greater anxiety symptoms were positively correlated with more autism traits and worse sleep overall, supporting previous findings of this relationship (Austin, 2005; Green and Ben-Sasson, 2010; Grondhuis and Aman, 2012). This suggests that anxiety may result in greater functional impact toward the high end of the spectrum of Neurodevelopmental symptoms. It can be expected that the increased experience of symptoms in this triad would be associated with greater social and behavioral impairments. Recognizing that adverse early childhood experiences can lead to difficulties in adolescence and adulthood, such as increased risk of suicidal behavior, highlights the importance of early intervention (Serafini et al., 2015).

The Obsessive Compulsive subscale was dominant across analyses of anxiety, sleep and autism trait measures, despite comprising only six out of fifty questions on the SCAS-P related to intrusive thoughts (e.g., the child experiences ‘bad’ or ‘silly’ thoughts) and subsequent behaviors (e.g., my child has to do things over and over again). The relationship of autism traits with obsessive compulsive behaviors across all AQ-Child subscales is reflective of a broader population comorbidity (van Steensel et al., 2011) that may be accounted for in part by shared pathophysiology and genetic factors (Meier et al., 2015). Although optimal levels of anxiety can be adaptive and increase performance (Arent and Landers, 2003), the chronicity of trait anxiety is reported to impact on overall biological functioning (Slattery et al., 2012).

The complexity of identifying factors associated with anxiety was exemplified by the high number of correlations identified across questionnaire total scores and subscales of the AQ-Child and SDSC with the SCAS-P questionnaire. Although total SCAS-P score was significantly correlated with total scores on both the AQ-Child and the SDSC and the SCAS-P as anticipated, these associations were not as pronounced as expected. While it could be considered that the AQ-Child and SCAS-P scales measure different constructs, we contend that anxiety and autism traits are inherently intertwined. However, the moderate relationship between anxiety and autism traits in the current sample may have been minimized due to our reliance on parent report.

### Limitations of Current Study and Future Research

The accuracy of parent reporting may have been influenced by a social desirability bias due to stigma or their own internal experiences. It can be speculated that parents completing the questionnaires may have had higher than average anxiety themselves due to a genetic predisposition to aspects of the Neurodevelopmental Phenotype and potential stressors of parenting a child with AHDN (Piven and Palmer, 1999; Ingersoll and Hambrick, 2011). As identified in previous research (Magiati et al., 2014), it is possible that parent ratings are not entirely reflective of their child’s anxiety due to a discrepancy between observable and internal experiences of anxiety attributed to difficulties in social skills, language ability and emotional expression in many children with AHDN.

The relationship between sleep behaviors and autism traits with anxiety in our correlational analyses suggest that using a sleep or autism behavior scale may be a useful screening tool to identify an anxious child. Autism traits and sleep difficulties are likely to be more overtly recognized by parents and subsequently easier to report in the given questionnaires, possibly because these behaviors could more explicitly demand the responsiveness of caregivers. For example, a parent attending to a child during the night when they cannot sleep or noticing their child has difficulty developing friendships may be easier to identify as areas of concern compared to physical symptoms of anxiety that could be attributed to various other factors such as tiredness or shyness.

Lastly it is also important to consider our findings within the context of the population used in this study. The current sample was characterized by a gender ratio, around two thirds male, that is representative of the broader gender disparities noted in Neurodevelopmental Disorders (World Health Organisation [WHO], 2013a). While our aim was to investigate factors contributing to an anxious child with other associated behaviors in children with AHDN, our findings are limited by the absence of a comparative typically developing sample. Though we did address this by comparing the current sample to published norms, future research would ideally examine differences in anxiety between children with AHDN and typically developing peers beyond past research. The understanding of childhood anxiety would be enhanced by incorporating child, teacher and parent ratings as well as objective biological measures such as blood pressure and heart rate. Future research should also further investigate the relationship of obsessive–compulsive behaviors with autism traits and sleep.

## Conclusion

The current study demonstrates the relationship between autism traits, sleep and anxiety in a sample of children experiencing difficulties at school. It provides evidence that autism-like behaviors and sleep difficulties may be identifiable expressions, and subsequently core biomarkers, of anxiety in children with AHDN. Although the ASD group had significantly higher anxiety compared to the Speech and Language group, the large range in scores across all groups highlights the importance of recognizing the functional impact of anxiety on behaviors for any individual with AHDN irrespective of diagnosis. Importantly, these children with additional needs demonstrated greater overall anxiety when compared to previously published data for normal controls, suggesting that anxiety is a common general experience in this population. Our results support the use of questionnaires as a conventional means of identifying behaviors in children in order to guide early intervention and inform treatment in the assessment of children experiencing social, emotional and academic difficulties. Although dual relationships between anxiety and autism-traits, as well as between anxiety and sleep have been demonstrated previously in primarily neurotypical populations, the current findings demonstrate that these interlinked relationships extend to children AHDN and may contribute to better characterization of these children earlier in childhood.

## Ethics Statement

This study was carried out in accordance with the recommendations of the La Trobe University Human Research Ethics Committee and the Victorian Department of Education Ethics Committee. All researchers had a government Working With Children’s Check. All subjects gave written and verbal informed consent in accordance with the Declaration of Helsinki.

## Author Contributions

AC was involved in the study design, research and development of theory, data collection, analysis and interpretation, and preparation of manuscript. NG assisted in data collection and interpretation, development of theory, and manuscript editing and review. RL contributed to the design of the study, data interpretation, development of theory, and manuscript editing and review. SC conceptualized the research design, contributed to theoretical and data interpretation, manuscript editing and review, and was the primary supervisor of the research study.

## Conflict of Interest Statement

The authors declare that the research was conducted in the absence of any commercial or financial relationships that could be construed as a potential conflict of interest.
